# Inhibition of *α*-Amylases by Condensed and Hydrolysable Tannins: Focus on Kinetics and Hypoglycemic Actions

**DOI:** 10.1155/2017/5724902

**Published:** 2017-05-14

**Authors:** Camila Gabriel Kato, Geferson de Almeida Gonçalves, Rosely Aparecida Peralta, Flavio Augusto Vicente Seixas, Anacharis Babeto de Sá-Nakanishi, Lívia Bracht, Jurandir Fernando Comar, Adelar Bracht, Rosane Marina Peralta

**Affiliations:** ^1^Postgraduate Program of Food Science, University of Maringá, Avenida Colombo 5790, 87020900 Maringá, PR, Brazil; ^2^Department of Biochemistry, University of Maringá, Maringá, PR, Brazil; ^3^Department of Chemistry, Federal University of Santa Catarina, Florianópolis, SC, Brazil

## Abstract

The aim of the present study was to compare the in vitro inhibitory effects on the salivary and pancreatic *α*-amylases and the in vivo hypoglycemic actions of the hydrolysable tannin from Chinese natural gall and the condensed tannin from* Acacia mearnsii. *The human salivary *α*-amylase was more strongly inhibited by the hydrolysable than by the condensed tannin, with the concentrations for 50% inhibition (IC_50_) being 47.0 and 285.4 *μ*M, respectively. The inhibitory capacities of both tannins on the pancreatic *α*-amylase were also different, with IC_50_ values being 141.1 *μ*M for the hydrolysable tannin and 248.1 *μ*M for the condensed tannin. The kinetics of the inhibition presented complex patterns in that for both inhibitors more than one molecule can bind simultaneously to either the free enzyme of the substrate-complexed enzyme (parabolic mixed inhibition). Both tannins were able to inhibit the intestinal starch absorption. Inhibition by the hydrolysable tannin was concentration-dependent, with 53% inhibition at the dose of 58.8 *μ*mol/kg and 88% inhibition at the dose of 294 *μ*mol/kg. For the condensed tannin, inhibition was not substantially different for doses between 124.4 *μ*mol/kg (49%) and 620 *μ*mol/kg (57%). It can be concluded that both tannins, but especially the hydrolysable one, could be useful in controlling the postprandial glycemic levels in diabetes.

## 1. Introduction

Both the pancreatic *α*-amylase and the salivary *α*-amylase (*α*-1,4-glucan-4-glucanohydrolase, EC 3.2.1.1) catalyse the hydrolysis of the *α*-1,4-glycosidic linkages in starch, glycogen, and other oligo- and polysaccharides. The salivary amylase (HSA), the most abundant enzyme in human saliva, initiates the digestion of complex carbohydrates in the human oral cavity, where especially starch is partly digested into oligosaccharides, maltose, and glucose [1]. The process is subsequently completed by the pancreatic *α*-amylase. In humans, five isoenzymes of *α*-amylase (*α*-1,4-glucan-4-glucanohydrolase, EC 3.2.1) have been described. The three isoforms of salivary *α*-amylase and the two isoforms of pancreatic *α*-amylase are classified as two different families of isoenzymes. The three-dimensional structures of the *α*-amylases from human pancreas and saliva and from porcine pancreas have already been determined by X-ray crystallography [2–4]. Structurally these enzymes are all very closely related. Due to its importance in several metabolic disorders including diabetes and obesity, the pancreatic *α*-amylase has been more extensively studied than the salivary *α*-amylase. In consequence, a series of pancreatic *α*-amylase inhibitors are available in the market, such as acarbose, voglibose, and miglitol [5–7]. The administration of these molecules can be a useful first-line treatment for diabetic patients who have a combination of slightly raised basal plasma glucose concentrations and marked postprandial hyperglycemia.

**Figure 1:**
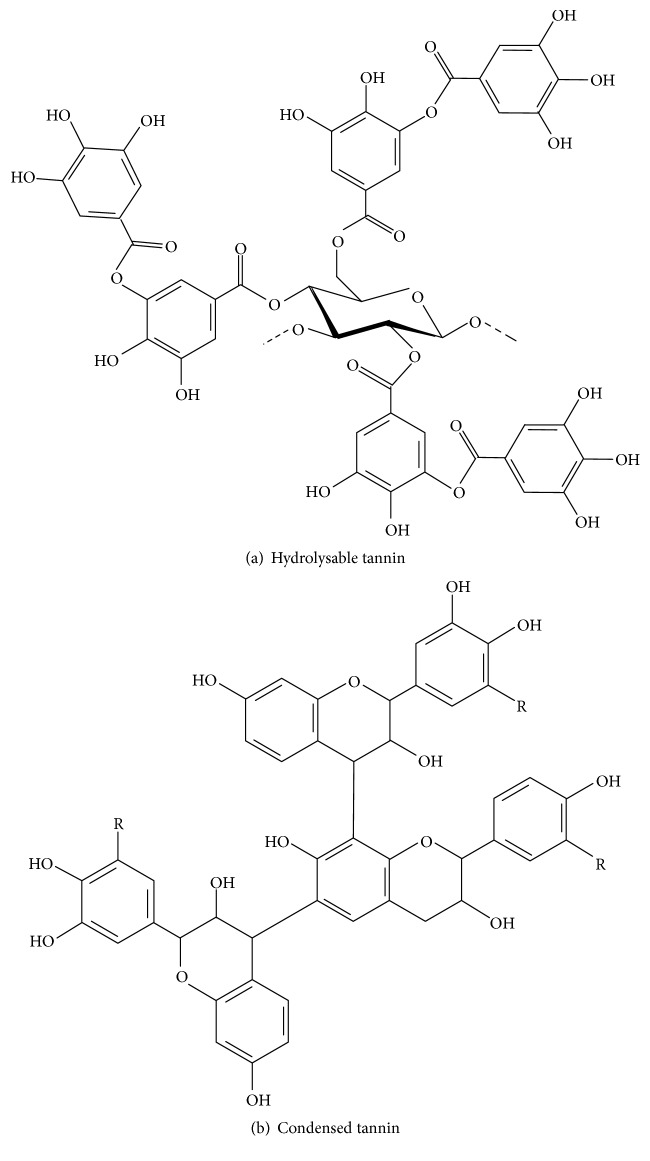
Repetitive structures of the hydrolysable tannin from Chinese natural gallnuts (tannic acid; mw 1701.2 g/mol [16]) and the condensed tannin from* A. mearnsii* (mw 806.0 g/mol [17]).

*α*-Amylase inhibitors help in the prevention and medical treatment of metabolic syndromes such as type 2 diabetes and obesity, in which they control the elevation of blood glucose levels by delaying and blocking postprandial carbohydrate digestion and absorption [8]. Different types of molecules were reported to possess *α*-amylase inhibitory activity. Among these molecules are flavonoids, polyphenolics, condensed tannins, hydrolysable tannins, terpenes, and cinnamic acid derivatives [9–12]. Tannins are naturally occurring plant polyphenols. Their main characteristic is that they bind proteins, basic compounds, pigments, large molecular weight compounds, and metallic ions and display antioxidant activities. They are amply distributed in nature and are present in fruits, teas, trees, and grasses. Hydrolysable tannins are derivatives of gallic acid (3,4,5‐trihydroxybenzoic acid). Gallic acid is esterified to a core polyol, and the galloyl groups may be further esterified or oxidatively cross-linked to yield more complex hydrolysable tannins. One of the most simple and common hydrolysable tannins is the gallotannin with up to 12 esterified galloyl groups and a core glucose (Figure 1(a)) as the gallotannin from Chinese natural gallnuts [13]. Condensed tannins are oligomeric and polymeric proanthocyanidins that can possess different interflavanyl coupling and substitution patterns [13, 14]. One of the most extensively studied proanthocyanidins is that one extracted from the bark of the black wattle tree* (Acacia mearnsii)*. It is rich in the catechin-like flavan-3-ols monomers robinetinidol and fisetinidol (Figure 1(b) [15]).

The aim of the present study is to compare the in vitro inhibitory effects of two tannins with well-known chemical structures on the salivary and pancreatic *α*-amylases and their putative in vivo hypoglycemic actions. The first one is the hydrolysable tannin from Chinese natural gall and the second one the condensed tannin from* A. mearnsii* (Figures 1(a) and 1(b)). In the in vitro experiments, especial attention has been devoted to the kinetics of the inhibition, with a detailed search for the model that best describes the mechanism of action.

## 2. Materials and Methods

### 2.1. Materials

Porcine pancreatic *α*-amylase (Type VI-B), human salivary *α*-amylase, acarbose, and the hydrolysable tannin from Chinese natural gallnuts were purchased from Sigma-Aldrich Co. The condensed tannin from* Acacia mearnsii *bark was purchased from Labsynth, Brazil.

### 2.2. Reaction Rate Measurements

The kinetic experiments with the porcine pancreatic *α*-amylase and the human salivary *α*-amylase were carried out at 37°C in 20 mmol/L phosphate buffer, pH 6.9, containing 6.7 mmol/L NaCl. Both temperature and pH of the assay are close to the optimum values reported in several studies. Potato starch (Sigma-Aldrich) was used as substrate. The substrate (0.05–1.0 g/100 mL) and one of the two inhibitors,* A. mearnsii *condensed tannin (up to 620 *μ*M) and hydrolysable tannin (tannic acid; up to 294 *μ*M), were mixed and the reaction was initiated by adding the enzyme. The specific activity of both enzymes was 500 units/mg protein. The amount of enzyme added to each reaction system was 1 unit. The reaction was allowed to proceed for 5 min. The reducing sugars resulting from the starch hydrolysis were assayed by the 3,5-dinitrosalicylic acid (DNS) method, using maltose as standard [18]. The aldehyde group of reducing sugars converts 3,5-dinitrosalicylic acid to 3-amino-5-nitrosalicylic acid, which is the reduced form of DNS. The formation of 3-amino-5-nitrosalicylic acid results in a change in the amount of light absorbed at 540 nm. The absorbance measured using a spectrophotometer is directly proportional to the amount of reducing sugar. The pH of the reaction medium was tested for all situations. No changes were detected during the incubation time.

### 2.3. Animal Experiments

Male healthy Wistar rats weighing 200–250 g were used in all experiments. The rats were housed, fed, and treated in accordance with the universally accepted guidelines for animal experimentation. Prior to the investigations, the animals were kept for one week under standard environmental conditions. Throughout the experimentation period, the rats were maintained in single cages and had access to standard pelleted diet and water ad libitum. Food was withdrawn 18 h before the experiments. All experiments involving rats were done in accordance with the worldwide accepted ethical guidelines for animal experimentation and previously approved by the Ethics Committee for Animal Experimentation of the University of Maringá (Protocol number 067/2014-CEUA-UEM).

### 2.4. Glycemic Levels of Rats after Starch Administration

Rats were divided into 9 groups (*n* = 4 rats per group). To group I (positive control) commercial corn starch (1 g per kg body weight) was given intragastrically. Group II (negative control) received only tap water. Group III (positive control) received intragastrically commercial corn starch plus acarbose (50 mg/kg). Groups IV, V, and VI received intragastrically commercial corn starch plus* A. mearnsii *tannin doses of 100, 250, and 500 mg/kg, respectively. Finally, groups VII, VIII, and IX received intragastrically commercial corn starch plus tannic acid doses of 100, 250, and 500 mg/kg, respectively. The amounts of inhibitors given to the rats were based on literature data [19]. Fasting blood glucose levels were determined before the administration of starch and amylase inhibitors (0 time). Later evaluations of blood glucose levels took place at 15, 30, 45, and 60 min. Blood glucose from cut tail tips was determined using Accu-Chek® Active Glucose Meter.

### 2.5. Calculations and Statistical Criteria

Statistical analysis of the data was done by means of the Statistica program (Statsoft, Inc., Tulsa, OK). Numerical interpolation for the determination of the half-maximal inhibitor concentrations (IC_50_) was done using the Scientist Software from MicroMath Scientific Software (Salt Lake City, UT). The same program was used for fitting the rate equations to the experimental initial rates by means of an iterative nonlinear least-squares procedure. The decision about the most adequate model (equation) was based on the model selection criterion (MSC) and on the standard deviations of the optimized parameters. The model selection criterion, which corresponds to the normalized Akaike Information Criterion [20], is defined as

(1) $$\begin{array}{c} MSC=\ln\left[\;\frac{\sum_{i=1}^{n}w_{i}{\left(Y_{{obs}_{i}}-{\overset{-}{Y}}_{obs}\right)}^{2}}{\sum_{i=1}^{n}w_{i}{\left(Y_{{obs}_{i}}-Y_{{cal}_{i}}\right)}^{2}}\;\right]-\frac{2p}{n}. \end{array}$$

*Y*_obs_ are the experimental reaction rates, ${\overset{-}{Y}}_{obs}$ is the mean experimental reaction rate, *Y*_cal_ is the theoretically calculated reaction rate, *w* is the weight of each experimental point, *n* is the number of observations, and *p* is the number of parameters of the set of equations. In the present work, the model with the largest MSC value was considered the most appropriate, provided that the estimated parameters were positive. When the MSC values differed by less than 5%, the mode yielding the smallest standard deviations for the estimated parameters was considered the most appropriate one.

## 3. Results

### 3.1. Concentration Dependence of the *α*-Amylases Inhibition

**Figure 2:**
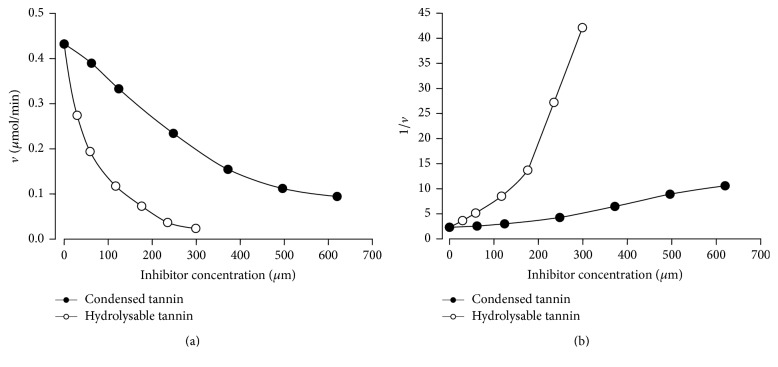
Inhibition of the human salivary *α*-amylase by condensed and hydrolysable tannins: concentration dependence. Initial reaction rates were measured as described in the Material and Methods. Each datum point represents the mean of four independent determinations. (a) Reaction rates (*v*); (b) inverse reaction rates (1/*v*).

Initially, inhibition of the activity of both *α*-amylases by the hydrolysable and condensed tannins was characterized in terms of the corresponding concentration dependence. For this purpose, initial rates were measured at a fixed starch concentration (1 g/100 mL) and variable tannin concentrations. Concentrations were expressed in *μ*mol/L (*μ*M), as the molecular weights of both tannins have already been determined [16, 17] and molar concentrations are much more informative about the number of molecules involved. The results of the experiments with the human salivary *α*-amylases are summarized in Figure 2. In Figure 2(a) the rates were represented against the inhibitor concentration. It is apparent that both condensed and hydrolysable tannins inhibited the enzyme with clear concentration dependence. From the graph it is also apparent that the hydrolysable tannin is a stronger inhibitor than the condensed tannin. IC_50_ value (the concentration of inhibitor required to reduce the rate of the enzymatic reaction by 50%) allows a quantitative evaluation of the effectiveness of each compound: it is equal to 47 *μ*M for the hydrolysable tannin and 285.4 *μ*M for the condensed tannin. This means that at the starch concentration of 1 g/100 mL the hydrolysable tannin is 6 times more effective as an inhibitor of the salivary *α*-amylase than the condensed tannin. In Figure 2(b) the reciprocals of the reaction rates (1/*v*) were represented against the corresponding concentrations. In both cases the relationship was parabolic even though this is less evident for the inhibition caused by the condensed tannin. This occurs because a single 1/*v* scale was used for both inhibitors and the inhibition degree with the hydrolysable tannin is much more pronounced, what causes a much more evident upward concavity.

**Figure 3:**
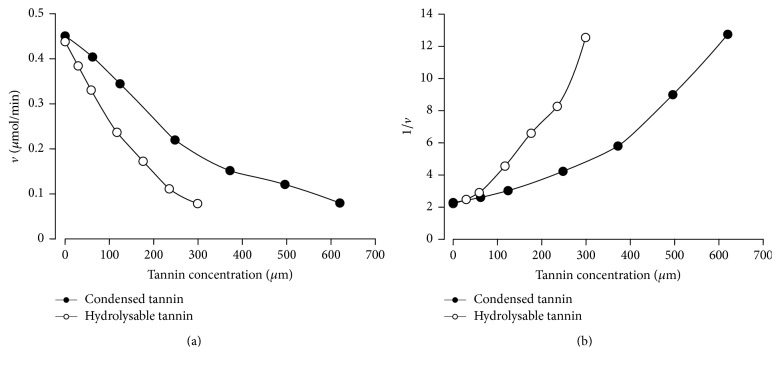
Concentration dependence of the porcine pancreatic *α*-amylase inhibition caused by the condensed and hydrolysable tannins: concentration dependence. Each datum point is the mean of three determinations. Reaction rates (*v*) and reciprocals of the reaction rates (1/*v*) were represented against the inhibitor concentrations.

The results of the measurements that were done with the porcine pancreatic *α*-amylase are shown in Figure 3. Both the hydrolysable and the condensed tannin inhibited the enzyme. The hydrolysable tannin was again more efficient, though the difference was less pronounced. In fact, IC_50_ for the hydrolysable tannin was 141.1 *μ*M and that for the condensed tannin 248.1 *μ*M. It is also noteworthy, because it has mechanistic implications, that the 1/*v* versus concentration plots revealed parabolic relationships for both inhibitors.

### 3.2. Kinetics of the Human Salivary *α*-Amylase Inhibition by the Hydrolysable and Condensed Tannins

**Figure 4:**
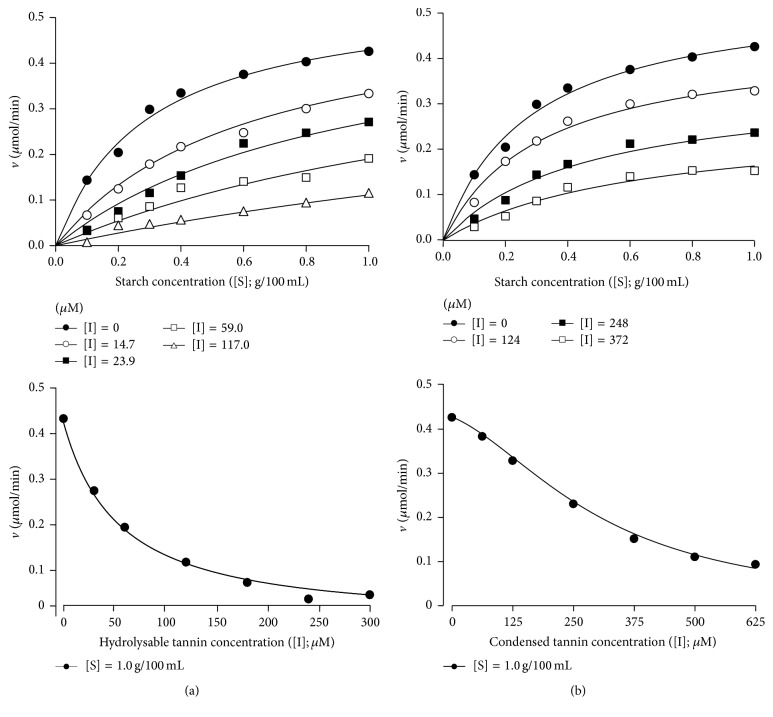
Reaction rates of the human salivary *α*-amylase obtained by varying simultaneously the substrate (starch) and the hydrolysable tannin (a) or condensed tannin (b) concentrations. Each datum point is the mean of four determinations. The lines running through the experimental points were calculated using optimized parameters obtained by fitting (3) ((a) hydrolysable tannin) or (4) ((b) condensed tannin) to the experimental data by means of a nonlinear least-squares procedure. Values of the optimized parameters and goodness-of-fit indicators for the hydrolysable tannin data (panel (a); equation (3)) are *K*_*M*_, 0.290 ± 0.027 g/100 mL; *V*_max_, 0.553 ± 0.018 *μ*mol/min; *K*_*i*1_, 13.2 ± 1.7 *μ*M; *K*_*i*2_, 343.4 ± 119.5 *μ*M; *K*_*i*3_, 609.6 ± 1086.2 *μ*M; sum of squared deviations, 0.00489; MSC, 4.535. For the condensed tannin data (panel (b); equation (4)) the optimized parameters are *K*_*M*_, 0.281 ± 0.024 g/100 mL; *V*_max_, 0.548 ± 0.016 *μ*mol/min; *K*_*i*1-2_, 194.9 ± 15.6 *μ*M; *K*_*i*3_, 705.9 ± 192.5 *μ*M; *K*_*i*4_, 369.6 ± 233.6 *μ*M; sum of squared deviations, 0.00381; MSC, 4.493.

When investigating the kinetic mechanism of the inhibitions caused by the hydrolysable and condensed tannins it is indispensable to take into account the form of the 1/*v* versus [I] plots shown in Figure 2. The parabolic relationships reveal that more than one inhibitor molecule can bind to at least one enzyme form [21, 22]. There are several mechanistic possibilities. The best way of investigating this is to measure the reaction rates by varying simultaneously the substrate concentration and the inhibitor concentration with subsequent model analysis in order to find out the mechanism that gives the best description of the experimental data. The results of the experiments that were done with the human salivary *α*-amylase are shown in Figure 4. Both the hydrolysable and the condensed tannins showed saturation curves that were progressively lowered as the tannins were added at progressively increased concentrations. The saturation curves do not show any tendency of convergence at high substrate concentrations, which excludes the possibility of competitive inhibition [21, 22]. Likewise, there is no decrease in the inhibition degree at low substrate concentrations, which would be indicative of uncompetitive inhibition [21, 22]. Most likely, thus, mixed (competitive-noncompetitive) inhibition must be considered in addition to the probability that at least two inhibitor molecules can bind to at least one form of the enzyme. The complete equation that applies to a mechanism in which the inhibitor binds twice and sequentially to the free enzyme (E) and to the enzyme-substrate complex (ES) is [22, 23]

(2) $$\begin{array}{c} v=\frac{V_{max}\left[S\right]}{K_{M}\left(1+\left[I\right]/K_{i1}+{\left[I\right]}^{2}/K_{i1}K_{i2}\right)+\left[S\right]\left(1+\left[I\right]/K_{i3}+{\left[I\right]}^{2}/K_{i3}K_{i4}\right)}. \end{array}$$

In (2), *V*_max_ is the maximal reaction rate, *K*_*M*_ the Michaelis-Menten constant, [S] the substrate concentration, and [I] the inhibitor concentration. The following inhibitory complexes are allowed: EI, EI_2_, ESI, and ESI_2_; *K*_*i*1_, *K*_*i*2_, *K*_*i*3_, and *K*_*i*4_ are the corresponding dissociation constants of these complexes (inhibitor constants). If one of these complexes is lacking, limiting forms of (2) will apply [21–23]. It must be noted that the squared inhibitor concentration ([I]^2^) accounts for the parabolic inhibition. Agreement between theory and experiment was tested by means of a least-squares fitting procedure. Fitting was done simultaneously with two independent variables ([S] and [I]), including the rate versus inhibitor concentration data shown in Figure 2. Attempts of fitting (2) to the set of data in Figure 4(a) (hydrolysable tannin) failed in that it was not possible to distinguish *K*_*i*3_ and *K*_*i*4_. This means that the enzyme-substrate complex (ES) forms only one type of complex with the inhibitor, which could be either ESI or ESI_2_. The latter implies in a simultaneous or almost simultaneous binding of two inhibitor molecules to the enzyme. After fitting the equations corresponding to 10 mechanistic possibilities, the best fit was achieved with (3), which describes the mechanism that allows the formation of complexes EI, EI_2_, and ESI:

(3) v=VmaxSKM1+I/Ki1+I2/Ki1Ki2+S1+I/Ki3.

All parameters have the meaning already described above. The continuous lines in Figure 4(a) represent the curves calculated by introducing the optimized parameters, given in the legend of Figure 4, into (3). It should be remarked that *K*_*i*1_ is much smaller than *K*_*i*2_ (26-fold). This means that the first binding of the hydrolysable tannin to the free enzyme occurs much more readily than the second one. Furthermore, *K*_*i*3_ is 46-fold higher than *K*_*i*1_, indicating that the complex ESI forms only at relatively high concentrations of the hydrolysable tannin. The legend of Figure 4 also gives the values of the sum of squared deviations and the model selection criterion (MSC), on which the decision about the most probable mechanism was based (see Materials and Methods). It should be stressed that (3) describes quite well both *v* versus [S] and *v* versus [I] curves (Figure 4(a)). Only at the highest [I] values a small systematic deviation was found, which could be indicating the existence of a small fraction of ESI_2_ complex.

Fitting of (2) to the data obtained with the condensed tannin (Figure 4(b)) was troubled by the impossibility of discriminating between *K*_*i*1_ and *K*_*i*2_. This could be indicating that the free enzyme (E) forms only one type of complex with the inhibitor, which could be EI or EI_2_. After fitting the equations corresponding to 10 mechanistic possibilities, the best fit was achieved with (4), which describes the mechanism that allows the formation of complexes EI_2_, ESI, and ESI_2_:

(4) v=VmaxSKM1+I2/Ki1-22+S1+I/Ki3+I2/Ki3Ki4.

*K*_*i*1-2_ is the dissociation constant for the complex EI_2_ (formed by the reaction E + 2I → EI_2_) and all other parameters have the meanings already specified above. In this particular case binding of two inhibitor molecules to the free enzyme occurs simultaneously or nearly so in such a way that the complex EI is practically absent. Comparison of theory and experiment in Figure 4(b) reveals a very good agreement, without systematic deviations at high inhibitor concentrations. Comparison of the numerical values of the inhibitor constants reveals stronger binding to the free enzyme (E) as *K*_*i*1-2_ is considerably smaller than *K*_*i*3_ by a factor of 3.6. Singularly, the second binding to the substrate-complexed enzyme (i.e., the formation of ESI_2_) is facilitated over the first one, as *K*_*i*4_ is smaller than *K*_*i*3_.

*K*_*M*_ and *V*_max_ values obtained when fitting (4) to the condensed tannin data and those obtained when (3) was fitted to the hydrolysable tannin data were practically the same, as given in the legend of Figure 4. This is actually expected because the data were obtained with the same enzyme, but agreement also speaks in favour of the correctness and reliability of the numerical analyses.

### 3.3. Kinetics of the Porcine Pancreatic *α*-Amylase Inhibition by the Hydrolysable and Condensed Tannins

**Figure 5:**
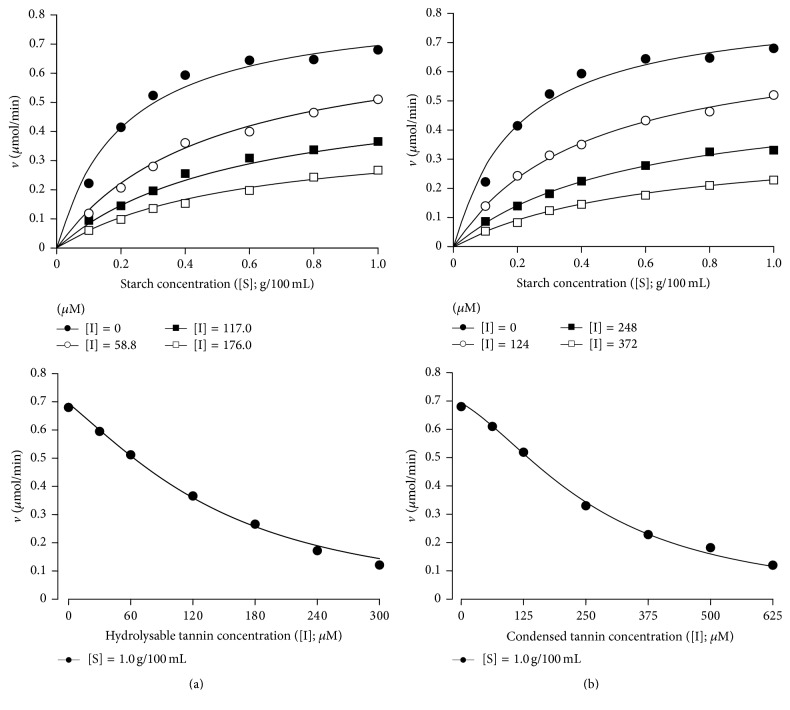
Reaction rates of the porcine pancreatic *α*-amylase measured by varying simultaneously the substrate (starch) and the hydrolysable tannin (a) or condensed tannin (b) concentrations. Each datum point is the mean of four determinations. The lines running through the experimental points were calculated using optimized parameters obtained by fitting (5) ((a) hydrolysable tannin) or (6) ((b) condensed tannin) to the experimental data by means of a nonlinear least-squares procedure. Values of the optimized parameters and goodness-of-fit indicators for the hydrolysable tannin data (panel (a); equation (5)) are *K*_*M*_, 0.207 ± 0.017 g/100 mL; *V*_max_, 0.839 ± 0.020 *μ*mol/min; *K*_*i*1_, 37.7 ± 3.7 *μ*M; *K*_*i*3-4_, 169.6 ± 10.6 *μ*M; sum of squared deviations, 0.0101; MSC, 4.545. For the condensed tannin data (panel (b); equation (6)) the optimized parameters are K_M_, 0.201 ± 0.015 g/100 mL; *V*_max_, 0.832 ± 0.018 *μ*mol/min; *K*_*i*1_, 147.0 ± 21.5 *μ*M; *K*_*i*2_, 663.2 ± 345.4 *μ*M; *K*_*i*3-4_, 441.6 ± 37.8 *μ*M; sum of squared deviations, 0.00786; MSC, 4.793.

The results of the experiments that were done with the hydrolysable tannin are shown in Figure 5(a). Simple inspection reveals many qualitative similarities to the inhibition caused by the hydrolysable tannin on the human salivary *α*-amylase (Figure 4(a)). In the search for the best mechanism that describes the set of data in Figure 5(a) the equations corresponding to 10 different mechanisms were fitted to the experimental data, including (2). Here again fitting of the complete (2) was unsuccessful and the best fitting was achieved with an equation that predicts the formation of complexes EI and ESI_2_:

(5) $$\begin{array}{c} v=\frac{V_{max}\left[S\right]}{K_{M}\left(1+\left[I\right]/K_{i1}\right)+\left[S\right]\left(1+{\left[I\right]}^{2}/{\left(K_{i3\text{-}4}\right)}^{2}\right)}. \end{array}$$

In (5), *K*_*i*3-4_ is the dissociation constant for the complex ESI_2_ (formed by the reaction ES + 2I → ESI_2_) and all other symbols have the same meanings specified above. The optimized parameters are listed in the legend of Figure 5. As can be deduced from the graphs in Figure 5(a), the calculated curves agree pretty well with the experimental ones with no systematic deviations at the extremes of both substrate and inhibitor concentrations. *K*_*i*1_ is 4.5 times smaller than *K*_*i*3-4_; the free enzyme, thus, binds much more strongly the hydrolysable tannin than the substrate-complexed enzyme.

The results of the kinetic investigations on the inhibition caused by the condensed tannin on the pancreatic *α*-amylase are shown in Figure 5(b). In this case, the best fit was found with an equation that allows the formation of complex EI in addition to the complexes EI_2_ and ESI_2_:

(6) v=VmaxSKM1+I/Ki1+I2/Ki1Ki2+S1+I2/Ki3-42.

*K*_*i*2_ is the inhibitor constant for the formation of complex EI_2_ from complex EI. Agreement between theory and experiment was as good as in the preceding analyses, as can be concluded by inspecting the graphs in Figure 5(b) and the statistical parameters in the legend of Figure 5. As in all preceding cases, formation of the EI complex is greatly favoured in comparison with the formation of all other complexes, as *K*_*i*3-4_ (ESI_2_) exceeds K_i1_ by a factor of 3.0 and *K*_*i*2_ (EI_2_) exceeds *K*_*i*1_ by a factor of 4.5.

### 3.4. In Vivo Inhibition of *α*-Amylase

**Figure 6:**
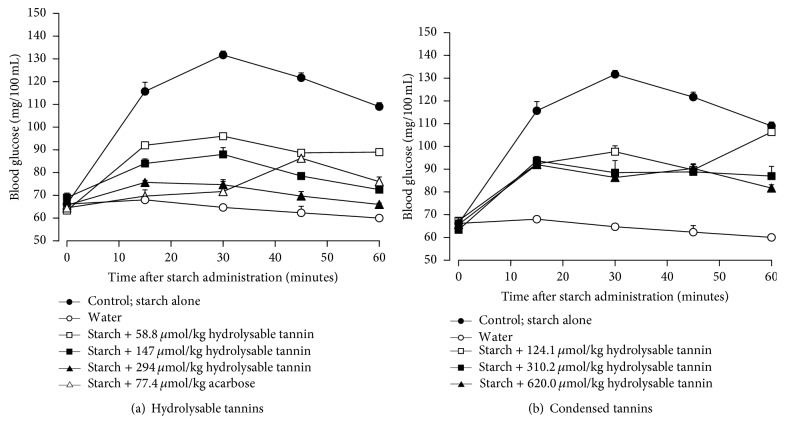
Influence of the hydrolysable tannins and acarbose administration on the glycemic levels of fasting rats during 60 min following starch administration. Blood samples from the tail vein were analyzed by means of a glucometer after intragastric starch administration (1 g per kg body weight). The hydrolysable and condensed tannins as well as acarbose were administered intragastrically at the doses given on the top. Each datum point represents the mean ± mean standard errors of three experiments. Experimental details are given in the Materials and Methods.

For testing in vivo the inhibition caused by both the hydrolysable and condensed tannin, starch was given to rats and the glycemic levels were followed during 60 minutes. The basis for these experiments is given by the well-established notion that hydrolysis of intragastrically administered starch is a prerequisite for the entrance of the derived glucosyl units into blood.  Figure 6(a) shows the time course of the experiments that were done by administering various doses of the hydrolysable tannin. When an aqueous solution of starch was administered alone the glycemic levels raised producing a concave down curve with a peak increment of 85% at 30 minutes after administration. When water was administered the glycemic levels remained relatively constant. Administration of starch in combination with various doses of hydrolysable tannin produced increases in the glycemic levels that were less pronounced than those found when starch was administered alone. A dose-dependent effect is apparent. In all cases, however, concave down curves were obtained. Starch plus acarbose administration, the positive control experiment, also diminished the increase in blood glucose concentration, especially during the first 30 minutes, with a peak at 45 minutes.

**Figure 7:**
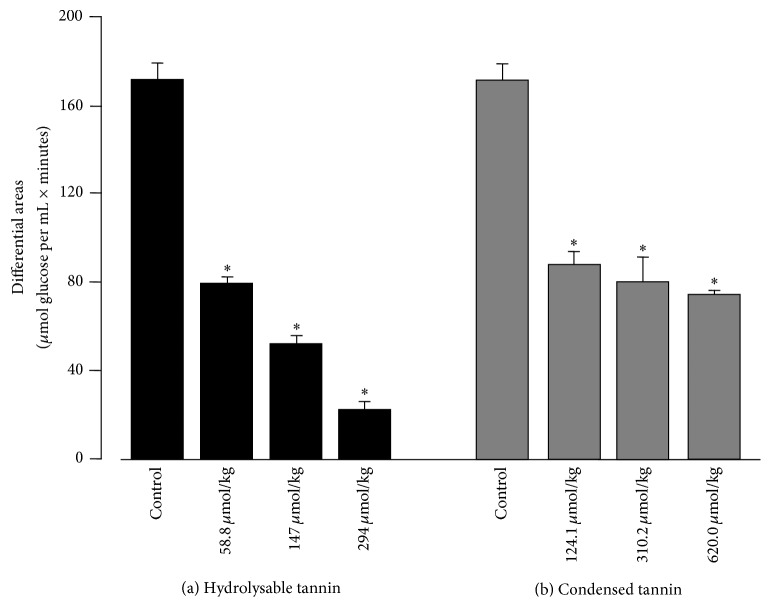
Areas between the glycemic curves after starch administration (starch alone or starch + *α*-amylase inhibitors) and the glycemic basal levels. The areas were determined with the corresponding data in Figures 6(a) and 6(b) using the numerical integration procedures of the Scientist Software from MicroMath Scientific Software (Salt Lake City, UT). The error terms correspond to standard errors of the means. Asterisks indicate statistical difference relative to the control experiment according to ANOVA followed by post hoc Student-Newman-Keuls testing (*p* ≤ 0.05).

Figure 6(b) shows the results of the experiments done with the condensed tannin. The control curves are the same shown in Figure 6(a). Coadministration of starch and condensed tannin resulted in diminished increases in the glycemic levels. However, the fivefold increase in the administered dose (124.1 to 620.0 *μ*mol/kg) did not result in a pronounced enhancement of the effect. This phenomenon can be best appreciated by comparing the areas under the glycemic curves in Figure 7. The areas were computed numerically and subtracted from the area under the curve obtained when water was administered alone. This area can be regarded as a measure of the extra glucose in the circulating blood during the first 60 minutes following starch and tannin administration. Figure 7 shows that the action of the hydrolysable tannin shows a well-defined dose-dependent action. The lowest dose already diminished the glycemic response by 53%; with the highest dose the diminution reached 88%. The action of the condensed tannin was similar to that of the hydrolysable tannin at the lowest dose (49%), but further increases in the administered dose were poorly effective, as the highest dose reduced the glycemic response by not more than 57%.

## 4. Discussion

Inhibition of the human salivary and porcine pancreatic *α*-amylases by both the hydrolysable and condensed tannins presents several complexities in that for both inhibitors more than one molecule can bind simultaneously to the enzymes [21, 22]. This is revealed a priori by the nonlinear 1/*v* versus [I] plots and confirmed by the numerical analysis in which attempts of fitting an equation describing linear inhibition (single binding) always produced unfavourable results. Even assuming some limited degree of heterogeneity for the preparations that were used, especially for the condensed tannin [17], it should be remarked that the phenomenon does not invalidate (2) or its limiting forms, provided that all concentrations are kept at constant ratios as it occurs when different amounts of the same preparation are added [21, 22]. In the latter case, however, the inhibition constants are no longer true dissociation constants but rather complex functions of several individual dissociation constants. They remain, notwithstanding, a measure of the potency of a given inhibitor [21–23]. Parabolic inhibition is a common phenomenon among phenolics and tannins. The inhibition of *α*-amylases by a* pinhão* coat tannin [24] and by the* Phaseolus *protein inhibitor*α*-AI [23] has been reported to be parabolic. Inhibition of the pancreatic lipase by a* pinhão* coat tannin is also of the parabolic type [25]. Furthermore, the fact that the same phenomenon occurs with a pure and well-defined substance such as acarbose, depending on the substrate [23, 26], is a proof that it is not generated by an eventual heterogeneity of the inhibitor. On the other hand, on some occasions the phenomenon has been neglected. For example, the inhibition of the human *α*-amylase by a gallotannin was analyzed as being of the linear type even though the Dixon plots (1/*v* versus [I]) that were presented are clearly indicating parabolic inhibition [27]. It should be noted that, in the experiments in which the substrate concentration was varied, the maximal tannin concentrations were smaller than those used in *v* versus [I] experiments. This occurred because it is difficult to measure accurately low initial reaction rates at low substrate concentrations. Even so, the description of *v* versus [I] relationships by the fitted equations was very good, with minimal deviations at the highest inhibitor concentrations. For all cases analyzed in the present work inhibition was of the mixed (competitive-noncompetitive) type. This is the most frequently reported mode of inhibition [23–27]. The longan pericarp proanthocyanidins, however, have been reported as a singular case of uncompetitive inhibition on the *α*-amylase [28].

By comparison of our data on both tannins with those on acarbose in the literature [23, 24, 26] it is obvious that acarbose is a much better inhibitor of the pancreatic *α*-amylase than the tannins. The question of which tannin is more effective can be unambiguously answered in the case of the human salivary *α*-amylase inhibition. The hydrolysable tannin concentration for half-maximal inhibition (IC_50_) of the salivary enzyme is considerably smaller than that of the condensed tannin (47 *μ*M compared to 285 *μ*M) and the same can be said about the tendency of binding to the free enzyme, which is 14.7 times superior in the case of the hydrolysable tannin. With respect to the pancreatic enzyme the difference is not as pronounced. In terms of their masses and at the substrate concentration of 1 g/100 mL the condensed tannin was a moderately better inhibitor because 50% inhibition occurred at the concentration of 141 *μ*M compared to the 248 *μ*M required for the same degree of inhibition by the hydrolysable tannin. However, the inhibition degree will vary with the substrate concentration and at low substrate concentrations the hydrolysable tannin will be a better inhibitor because of the smaller value of its inhibition constant *K*_*i*1_ (see legend of Figure 5).

A characteristic of the inhibitory action of both the hydrolysable and the condensed tannin is the observation that they are bound more tightly by the free enzyme (E) than by the enzyme complexed with the substrate (SE). This is revealed by the observation that for both enzymes *K*_*i*1_ (or *K*_*i*-12_) values for the hydrolysable and the condensed tannins were always considerably smaller than *K*_*i*3_ or *K*_*i*3-4_ values. This suggests that binding of the substrate, which is a large molecule, to the enzyme promotes structural modifications that make binding of the inhibitor more difficult in the case of the tannins.

Both hydrolysable and condensed tannins are well-known protein precipitating agents. Two different models have been suggested for explaining the capability of tannins to precipitate proteins [29]. It has been proposed that nonpolar tannins, such as pentagalloyl glucose, form a hydrophobic coat around the proteins, whereas polar tannins, such as epicatechin_16_ (4 → 8), form hydrogen-bonded cross-links between the protein molecules [29]. However, precipitation of proteins requires high concentrations of tannins. In the present and other studies, inhibition of the human salivary *α*-amylase by both the hydrolysable and the condensed tannin occurred in the presence of relatively low tannin concentrations, implying that the inhibitory action is due to specific molecular interactions involving specific amino acid residues of the protein and well-defined structural parts of the tannins. Binding of tannins to proteins have been demonstrated by methods that do not involve kinetics and they have been investigated for several combinations of tannins and proteins, including enzymes [30–32]. It can be deduced from these studies that binding of tannins to proteins involves both hydrophilic and hydrophobic interactions. It can be nonspecific in some cases and specific with a certain degree of cooperation in others [30]. In the case of the hydrolysable and condensed tannins used in the present study, binding is certainly a complex phenomenon, as indicated by the parabolic inhibition kinetics and probably facilitated by the numerous hydroxyl groups present in these molecules (see Figure 1). These groups could be especially important for the interactions at low concentrations [31]. The higher density of these groups on the hydrolysable tannin could be an explanation for its higher degrees of inhibition at low concentrations, especially with respect to the human salivary *α*-amylase. Consistent with this is the fact that hydroxylation of flavonoids improves the inhibitory effect on *α*-amylase exerted by these compounds [33]. At high concentrations, however, random hydrophobic stacking of the planar aromatic rings may occur between tannin and protein [30]. In this context, based on data from surface plasmon resonance binding experiments and nuclear magnetic resonance analyses [34] it has been proposed that the inhibitory effect caused by pentagalloyl glucose on the human salivary *α*-amylase results from the interaction of aromatic rings of the former with aromatic amino acids of the protein. The role of the aromatic amino acids of the human salivary *α*-amylase in the pentagalloyl glucose binding was reinforced by kinetic studies with W58L and Y151M mutants of the enzyme: replacement of the aromatic amino acids in the active site by aliphatic ones decreased inhibition dramatically, what seems to be in accordance with a participation of these residues in the interaction of tannins with the human salivary *α*-amylase [34].

Inhibition of *α*-amylase (as well as *α*-glucosidase) resulted in delayed carbohydrate digestion and glucose absorption with attenuation of postprandial hyperglycemic excursions. The diminution of hyperglycemia in rats to which starch was given by both the hydrolysable and condensed tannin is thus an expected phenomenon. Demonstration of the phenomenon also proofs that both tannins are able to exert *α*-amylase inhibition under in vivo conditions and not only in the test tube. In spite of the relatively small difference in their inhibitory activities toward the enzyme, the hydrolysable tannin was more effective in lowering hyperglycemia when compared to the condensed tannin. The response practically ceased to increase with condensed tannin doses above 124.1 *μ*mol/kg (100 mg/kg), whereas the effects of the hydrolysable tannin increased progressively until the dose of 620 *μ*mol/kg. The reasons for this behavior are not clear. One possible reason is that the hydrolysable tannin is active on other enzymes equally involved in starch digestion as the *α*-glucosidases and invertases, for example, whereas the condensed tannin is inactive or less active [19, 35]. Such a phenomenon would enhance the effectiveness of the hydrolysable tannin. Another possible reason, which does not exclude the former, is that the two types of tannins could be suffering the consequence of different gastric events and movements able to affect their effectiveness as inhibitors.

Besides participating in the initial hydrolysis of starch and other carbohydrate constituents of the diet, the salivary *α*-amylase exerts two additional functions, namely, binding to the tooth surface and binding to oral streptococci [36–38]. All three actions contribute to the process of dental plaque and caries formation. Binding to the enzyme is likely to restrict these three activities. A number of studies have shown that tea extracts (*Camellia sinensis* (L.) Kuntze) reduce dental caries [39, 40]. Based on the *α*-amylase inhibitory activity of tea extracts, the hypothesis has been raised that this activity could be involved in the reduction of the cariogenicity of starch-containing foods [41, 42]. For this reason, the hydrolysable tannin, which is bound very strongly by the enzyme, as indicated by *K*_*i*1_ value of 13.2 *μ*M, can be regarded as a useful agent for oral health.

## 5. Conclusion

In conclusion, both tannins are potentially useful in controlling the postprandial glycemic levels in diabetic patients, with the hydrolysable one, however, being superior. To our knowledge, the present study is the first one that presents a comparison of the effects of both types of tannins under exactly the same conditions. Clinical studies are evidently indispensable for evaluating the viability and safety of the use of preparations containing the hydrolysable or even condensed tannins, especially as food additives. With reference to the latter, more studies are also necessary to evaluate the possibility of incorporating this tannin into dental products such as dentifrices, mouthwashes, dental flosses, and chewing gums that could be helpful in the prevention of dental caries.
